# Baicalein Enhances Longevity and Healthspan of *C. elegans* Through the Insulin/IGF‐1 Signaling Pathway

**DOI:** 10.1002/mco2.70543

**Published:** 2025-12-17

**Authors:** Chen Zhao, Daniel Schrapel, Michael Schaefer

**Affiliations:** ^1^ Department of Pediatrics Union Hospital Tongji Medical College Huazhong University of Science and Technology Wuhan China; ^2^ Molecular OncoSurgery, Section Surgical Research Department of General Visceral & Transplant Surgery University of Heidelberg Heidelberg Germany

**Keywords:** aging, baicalein, *Caenorhabditis elegans*, DAF‐2, DAF‐16

## Abstract

Baicalein, a bioactive flavonoid derived from *Scutellaria baicalensis*, possesses notable anti‐inflammatory, antioxidative, and anticancer properties. Despite its therapeutic potential, the full scope of its effects on healthspan and longevity remains unexplored. This study investigates the impact of baicalein on longevity and health‐related biomarkers using the nematode *Caenorhabditis elegans*. Baicalein was administered to a wild‐type N2 strain, seven mutant strains, and three reporter strains. Its influence on longevity, motility, lipofuscin accumulation, and oxidative stress resistance was assessed. The methodology included Kaplan–Meier survival analysis, in vivo imaging, fluorescence microscopy, and real‐time PCR to evaluate RNA and protein expression. The findings indicate that baicalein significantly extends lifespan and enhances health markers, including improved motility, increased oxidative stress resistance, and reduced lipofuscin accumulation. Mechanistically, baicalein suppressed the DAF‐2‐mediated insulin/IGF‐1 signaling pathway and promoted the nuclear translocation of DAF‐16, a pivotal longevity transcription factor. Furthermore, baicalein upregulated the expression of the *sod‐3* gene, which is associated with enhanced stress tolerance and lifespan extension. These results elucidate the function of baicalein in promoting longevity and healthspan in *C. elegans* through modulation of insulin/IGF‐1 signaling. Future studies are warranted to explore the applicability of baicalein in human aging to pave the way for innovative antiaging supplement formulations.

## Graphical abstract

**Diagram showing how baicalein mediates lifespan signaling**. The phosphorylation of the transcription factor DAF‐16 is inhibited when the normal insulin/insulin‐like growth factor 1 signaling pathway is disturbed. The *daf‐16* nucleus translocation, made possible by this dephosphorylation, to start the *sod‐3* transcription that confers longevity, metabolic efficiency, and stress tolerance, is thought to help *C. elegans* live longer.

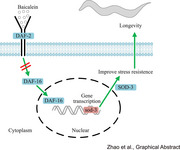

## 1 Introduction

Modern societies have achieved significant lifespan extension through improved public health policies. Yet, aging populations face heightened vulnerability to chronic diseases [1, 2]. While scientific progress enhances longevity, accumulated cellular and tissue degeneration progressively compromises bodily functions [3, 4]. This biological reality drives growing research into aging mechanisms to develop effective interventions against age‐related decline.

**FIGURE 1:**
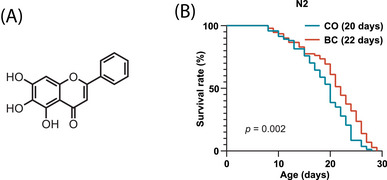
Baicalein extends lifespan. (A) Chemical structure of 5, 6, 7‐trihydroxyflavone baicalein. (B) Synchronized L1 wild‐type N2 were maintained on NGM/OP50 plates containing 100 µM baicalein (BC) or 0.1% DMSO (CO) for lifespan analysis. Representative lifespan data are shown (*p* value and median survival in parentheses; *p *< 0.05 indicates significance).

Natural polyphenols (e.g., curcumin [5]), dendrobium nobile alcohol extracts [6], alkaloids (berberine [7]) and organosulfur compounds (sulforaphane [8]) activate antioxidant pathways to combat aging, which can offer strategies to extend healthspan and reduce age‐related diseases. Notably, baicalein, a key flavonoid in *Scutellaria baicalensis* (*S. baicalensis*) roots, contains hydroxyl groups on its flavone structure that confer potent antioxidant activity [9, 10] (Figure 1A). Baicalein has exhibited multitarget antiaging potential through synergistic antioxidant [10] and anti‐inflammatory actions [11], neuroprotection [12], and cardiometabolic regulation [13]. Preclinical studies indicate its ability to neutralize oxidative stress via forkhead box protein (FOXO) pathway activation including upregulating superoxide dismutase (SOD) and catalase [14], to reduce amyloid‐beta accumulation linked to neurodegeneration [12], and to inhibit cancer progression through apoptosis induction [15]. Concurrently, it improves lipid metabolism and mitigates atherosclerosis, which addresses key aging drivers [13]. These integrated effects position baicalein as a promising therapeutic candidate for extending healthspan. However, its pleiotropic mechanisms require further elucidation.

The soil nematode *C. elegans* has been a popular and cornerstone model for studying the aging process [16]. So far, the lifespan extension effects of baicalein have been characterized to some extent using *C. elegans* model [17, 18, 19]. And as a pivotal breakthrough in aging research, the insulin/insulin‐like growth factor 1 (IGF‐1) signaling (IIS) pathway has been found to modulate lifespan in *C*. elegans [20]. This evolutionarily conserved pathway governs development, metabolism, and longevity across species. To put it more profoundly, DAF‐2 (the homolog of mammalian insulin/IGF‐1 receptor) triggers a phosphorylation cascade upon activation by insulin‐like peptides, thereby suppressing DAF‐16 (the homolog of mammalian FOXO). This suppression blocks DAF‐16 nuclear translocation and its subsequent activation of stress‐response genes. These two components function antagonistically: while DAF‐2 promotes growth at the expense of lifespan, DAF‐16 enhances stress resistance and longevity by upregulating genes such as *gst‐4*, *sod‐3*, and *mtl‐*1 [8]. Despite of recent research efforts devoted to baicalein's effects on longevity of *C. elegans*, several limitations exist in these studies, such as ignoring the dietary restriction‐induced longevity [17], insufficient lifespan assays without experimental repetitions [18], and performing lifespan assays with only N2 wild‐type strain and no necessary mutants for verification [19]. Therefore, well‐designed experiments and convincing results are urgently needed to reveal the mechanisms underlying baicalein‐induced longevity in *C. elegans*.

In this study, we carried out a series of delicately designed experiments to dissect the antiaging properties of baicalein using *C. elegans* as a biological model. Besides, the limitations in existing studies were also addressed to provide more convincing results and solid findings. First, does the potential factor, dietary restriction‐induced longevity, confound the lifespan‐extending effects of baicalein? Second, how does baicalein improve age‐related physiological functions and enhance healthspan? Third, what molecular mechanisms mediate the effects of baicalein on stress resistance and longevity? With extensive lifespan assays using seven mutant strains and three reporter strains, our studies reveal that baicalein significantly extends the lifespan of *C. elegans* and delays aging‐related phenotypic changes. This is achieved by inhibiting DAF‐2 (insulin/IGF‐1 receptor) signaling and facilitating DAF‐16 nuclear translocation, which leads to increased expression of the longevity‐associated gene *sod‐3*. These findings underscore the potential of baicalein as a focus of antiaging research.

## 2 Results

### 2.1 Baicalein Extends the Lifespan of Wild‐Type N2 *C. elegans*

As shown in Figure 1B, baicalein extended the median lifespan of wild‐type N2 *C. elegans* to 22 days (vs. 20 days in controls). And the results of three independent experiments indicated a 105–115% lifespan prolongation with statistical significance (all *p *< 0.05;) as shown in Table S1, which implies the marked longevity‐enhancing effects of baicalein.

### 2.2 Baicalein Has no Effect on *C. elegans* Feeding Preference or Growth of *E. coli* OP50

**FIGURE 2:**
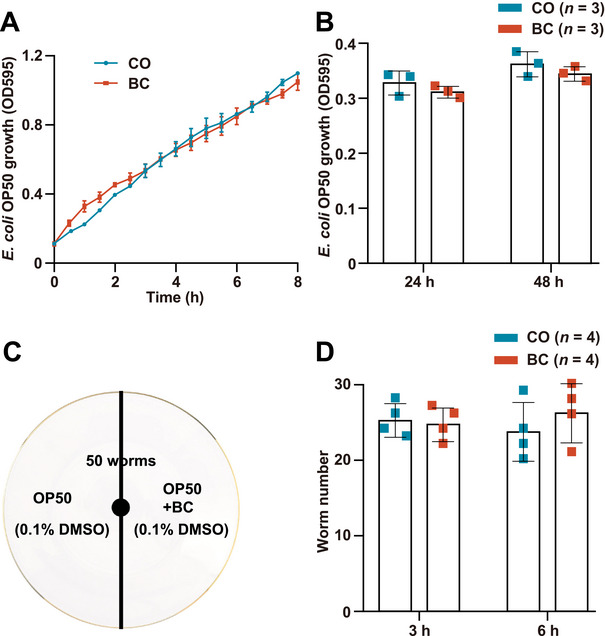
Baicalein does not inhibit the growth of *E. coli* OP50 food bacteria or affect food preferences of *C. elegans*. (A) *E. coli* OP50 growth was tested for BC and CO groups. OD_595_ readings were taken every 30 min from 0 to 8 h (three replicates, *p *> 0.05). (B) Extended monitoring at 24/48 h (three replicates, *p *> 0.05). (C) On a 6 cm diameter NGM agar plate, *E. coli* OP50 was seeded on the respective halves of the plate as indicated. Fifty nematodes were placed in the center and tracked at 3/6 h. (D) Migration counts mean ± standard deviation (SD), four replicates, *p* > 0.05.

To assess potential dietary restriction effects via *Escherichia coli* (*E. coli*) OP50 growth inhibition by 100 µM baicalein [21, 22], bacterial growth was monitored over 8 h (30‐min intervals) and at 24/48 h. Both baicalein‐ and 0.1% dimethyl sulfoxide (DMSO)‐treated groups showed comparable growth trajectories (Figure 2A, B), confirming no antibacterial activity.

A bacterial avoidance assay (Figure 2D) using NGM plates with 0.1% DMSO‐ versus baicalein‐treated *E. coli* OP50 revealed no significant differences in *C. elegans* distribution between the two halves of the plate at 3/6 h, indicating unchanged feeding preference.

### 2.3 Baicalein Improves Mobility, Lipofuscin Accumulation, and Stress Resistance in Aged Wild‐Type N2 *C. elegans*

**FIGURE 3:**
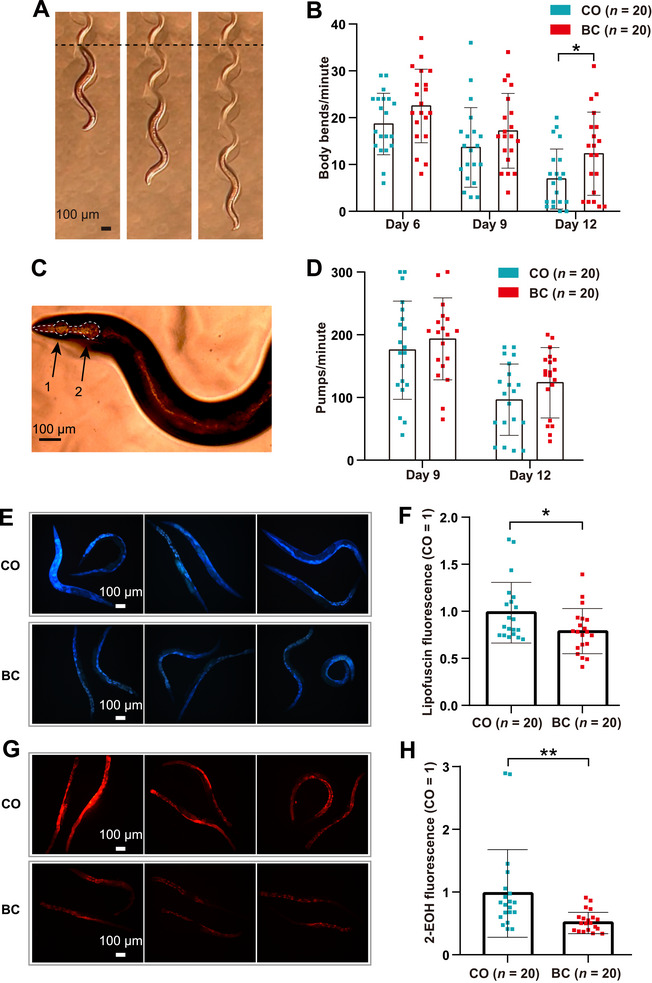
Baicalein improves healthspan, reduces lipofuscin accumulation, and enhances stress resistance in aged wild‐type N2 *C. elegans*. Age‐synchronized L1 of wild‐type N2 nematodes were cultured for BC and CO groups. (A) Body bending (representative images). (B) Body bends/minute (Days 6/9/12; *n* = 20) in mean ± SD, *p < 0.05. (C) Pharyngeal zones: 1 (meta corpus), 2 (terminal bulb). (D) Pharyngeal pumping (Days 9/12; *n* = 20; mean ± SD, *p* > 0.05). (E) Day 12 lipofuscin fluorescence (*n* = 20; three representative images). (F) Lipofuscin intensity (normalized to CO; mean ± SD, *p < 0.05). (G) Day 10 ROS‐induced fluorescence (*n* = 20; three representative images). (H) ROS intensity (normalized to CO; mean ± SD, **p < 0.01).

To evaluate the effect of baicalein on healthspan in *C. elegans*, aged N2 nematodes underwent body bending assays (Days 6/9/12). Baicalein‐treated groups showed increased bending frequency, particularly on Day 12 (Figure 3A, B), which indicated enhanced mobility. Pharyngeal pumping, measured via meta corpus/terminal bulb contractions (Figure 3C), showed no significant difference versus controls (Figure 3D), which suggested selective neuromuscular improvement. Lipofuscin assays quantified lysosomal age‐pigment accumulation (a biomarker of aging [23]) in 12‐day *C. elegans*. Imaging (Figure 3E) and fluorescence intensity analysis (Figure 3F) revealed significantly reduced lipofuscin levels in baicalein‐treated nematodes, which indicated attenuated biological aging. Dihydroethidium (DHE)/2‐hydroxyethidium (2‐EOH) assays measured superoxide levels via oxidative stress marker 2‐EOH (red fluorescence) [8]. Imaging (Figure 3G) and quantification (Figure 3H) demonstrated significantly reduced fluorescence in baicalein‐treated *C. elegans*, which indicated reactive oxygen species (ROS) and oxidative stress mitigation.

### 2.4 Investigation of Key Pathways Underlying Baicalein‐Induced Longevity

**FIGURE 4:**
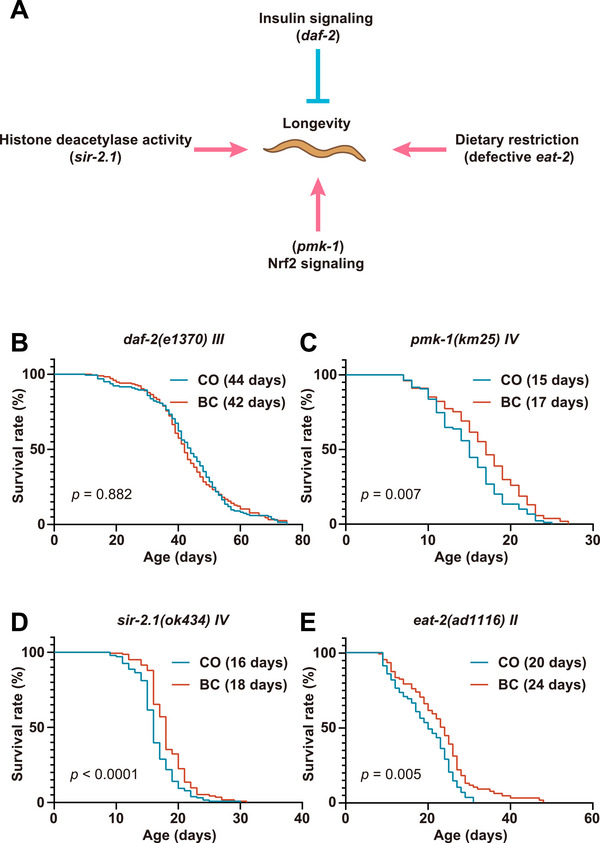
Baicalein extends *C. elegans* lifespan via DAF‐2 insulin/IGF‐1 signaling. (A) *C. elegans* longevity pathways schematic highlighting key genes. (B–E) Lifespan analysis of *daf‐2* (B), *pmk‐1* (C), *sir‐2.1* (D), and *eat‐2* (E) mutants for BC and CO groups. Representative survival curves are shown with median lifespan and *p* values (*p* < 0.05 indicates significance).

The mechanisms of lifespan extension of baicalein in *C. elegans* were explored through key longevity pathways (Figure 4A), which included the IIS pathway [24], *pmk‐1*/p38 MAPK‐regulated Nrf2 oxidative stress response [25], *sir‐2.1*‐dependent histone deacetylase activity [26], and *eat‐2*‐mediated dietary restriction [27]. Notably, administration of 100 µM baicalein significantly extended the median lifespan of *pmk‐1(km25)* mutants, *sir‐2.1(ok434)* mutants, and *eat‐2(ad1116)* mutants compared with their respective controls. However, it failed to extend the lifespan of *daf‐2(e1370)* mutants (Figure 4B–E). These results strongly implicate the DAF‐2/IIS pathway as a critical mediator of baicalein‐induced longevity, while its action is independent of *pmk‐1*‐mediated oxidative stress resistance, *sir‐2.1*‐dependent histone deacetylase activity, or *eat‐2*‐mediated mimicry of dietary restriction. The results of lifespan assays are shown in Table S2.

### 2.5 Aging and Stress Resistance Induced by Baicalein are Mediated by DAF‐16/FOXO

**FIGURE 5:**
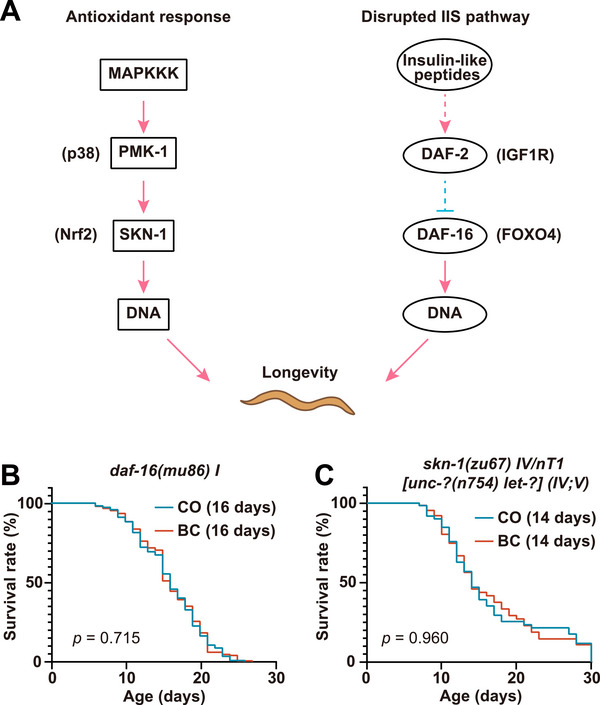
Baicalein promotes longevity via *daf‐16* and *skn‐1* signaling. (A) *C. elegans* longevity signaling pathways schematic (*skn‐1*, *daf‐16* highlighted). *daf‐16* (B) and *skn‐1* (C) mutants were maintained on NGM agar plates with *E. coli* OP50 for BC and CO groups for lifespan analysis. Representative lifespan curves show median survival and *p* values (*p* > 0.05).

IIS pathway, an evolutionarily conserved longevity mechanism from yeast to mammalian systems in various contexts, has well‐characterized upstream and downstream cascades [16, 20]. To further investigate the pro‐longevity mechanisms of baicalein in *C. elegans*, we focused on DAF‐16 (downstream of DAF‐2) and the Nrf2 ortholog SKN‐1 (downstream of PMK‐1) (Figure 5A). The Lifespans assay of *daf‐16(mu86)* mutants and *skn‐1(zu67)* mutants showed no significant lifespan changes with baicalein treatment (Figure 5B, C and Table S2), which confirmed the essential role of DAF‐16 and the necessity of SKN‐1 for baicalein‐induced longevity.

### 2.6 Baicalein Upregulates SOD‐3 Expression Following DAF‐16 Nuclear Translocation

**FIGURE 6:**
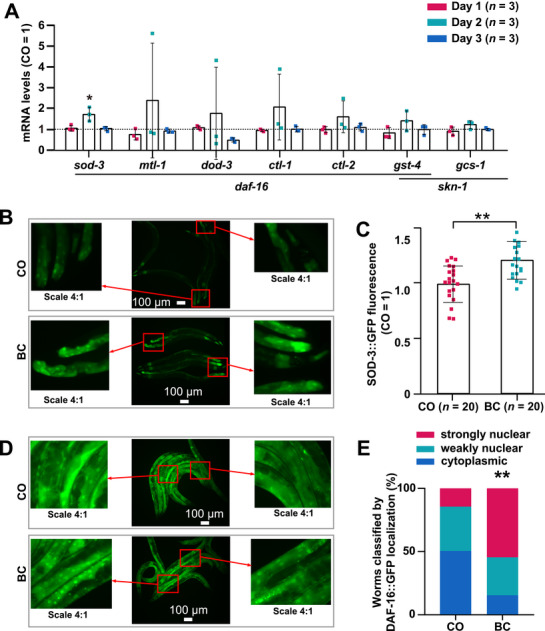
DAF‐16 mediates baicalein‐induced longevity and stress resistance. (A) Age‐synchronized L1 of wild‐type N2 were cultured for BC and CO groups. Total RNA from 200 nematodes/group (adult Days 1–3) was analyzed by real‐time polymerase chain reaction (PCR) for *sod‐3*, *mtl‐1*, *dod‐3*, *gst‐4*, *ctl‐1*, *ctl‐2*, *gcs‐1* (normalized to *act‐1*). Control standardized to 1 (dashed line); data as mean ± SD (**p < 0.05). (B) CF1553 (*sod‐3::gfp*) L1 treated with BC or CO (*n* = 20). Day 2 GFP captured (three representative images). (C) Fluorescence intensity quantified (normalized to CO; mean ± SD, **p < 0.01). (D) Age‐synchronized TJ356 (*daf‐16::gfp*) were treated with BC or CO (*n* = 20). Day 2 GFP captured (three representative images). (E) DAF‐16::GFP nuclear localization graded by n/c ratio: >2.0 (nuclear), 1.2–2.0 (weak), <1.2 (cytoplasmic). Data were analyzed by Fisher's exact test (**p < 0.01).

To investigate how baicalein activates DAF‐16 and SKN‐1, real‐time polymerase chain reaction (PCR) was used to analyze their downstream targets. DAF‐16‐regulated genes included *sod‐3*, *mtl‐1*, *dod‐3*, *gst‐4*, *ctl‐1*, and *ctl‐2*, while SKN‐1 primarily targeted *gst‐4* and *gcs‐1* [8, 28]. Our results showed that the expression of *sod‐3* significantly increased in the baicalein‐treated group on adult Day 2 compared with the control group. In contrast, the expression levels of other genes were not significantly improved at any time point compared with their respective controls (Figure 6A).

To determine if baicalein promotes DAF‐16 nuclear translocation and enhances SOD‐3 expression, the *sod‐3*::*gfp* reporter strain CF1553 driven by the *daf‐16* promoter was analyzed. Green fluorescent protein (GFP) fluorescence imaging on adult Day 2 (representative images in Figure 6B) and quantification (Figure 6C) showed that baicalein significantly increased SOD‐3::GFP fusion protein levels, which confirmed DAF‐16 activation and subsequent SOD‐3 upregulation.

To evaluate whether baicalein influences DAF‐16 nuclear translocation and its subsequent transcription activity, the TJ356 strain, which carries a *daf‐16*::*gfp* reporter construct, was treated with 100 µM baicalein or 0.1% DMSO. On the second day of adulthood, GFP fluorescence was measured using fluorescence microscopy. Representative images are shown in Figure 6D and nuclear translocation scores were calculated in Figure 6E. The presence of green foci indicated a significant increase in nuclear GFP fluorescence in the baicalein‐treated group, although there was no noticeable difference in overall fluorescence intensity, which is indicative of increased DAF‐16 transcription activity. In contrast, the GFP fluorescence activity was insufficient to provide conclusive evidence of SKN‐1 translocation, as shown in Figure S1.

## 3 Discussion

Our study employed *C. elegans* to investigate the antiaging effects of dietary flavonoid baicalein. Results showed baicalein extended lifespan and improved healthspan primarily via the IIS pathway. Baicalein inhibited insulin receptor‐like DAF‐2 and activated DAF‐16/FOXO, which upregulated *sod‐3* to enhance stress resistance and longevity.

Baicalein exhibited a lifespan extension of 105–115% and enhanced healthspan, as demonstrated by improved mobility, attenuated lipofuscin accumulation, and reduced ROS levels. Since this study chose 100 µM baicalein as good start point reported by Havermann et al. [18], future work should explore dose‐dependent effects for maximal lifespan benefits. While pharyngeal pumping activity remained unaltered, which is a phenotype consistent with its previously characterized selective neuromuscular modulation [18]. Crucially, lifespan assays in *eat‐2* mutants revealed that the *eat‐2* is dispensable for baicalein‐induced longevity, which can provide genetic evidence that further validates the pharyngeal pumping results and excludes canonical dietary restriction pathways in its mechanism.

Unlike quercetin and resveratrol in nematode lifespan regulation, baicalein demonstrates distinct advantages. 10–20 µmol/L quercetin can promote apoptotic cell death rather than mitigating cellular senescence in senescent human preadipocytes. Still, in vivo effects of quercetin across mammalian models remain unelucidated [29]. And the in vivo effects of quercetin on rodents or non‐human primates need further investigation. Resveratrol extends lifespan by inducing autophagy in a Sirt1‐dependent manner and requires NAD^+^ levels to support [30].

Our study employed live *E. coli* OP50 prepared using the LB medium method described previously for the lifespan assays [31]. As baicalein may undergo bacterial metabolic transformations (e.g., hydrogenation, ring‐fission), the observed lifespan extension in *C. elegans* could arise from baicalein itself, its bacterial metabolites, or their combined effects. A key limitation is our inability to distinguish whether the effect is driven by baicalein itself, its metabolites, or their combined action. Bacterial metabolism was not the primary focus of our investigation. Future studies should evaluate the longevity effects of purified bacterial metabolites to clarify their specific roles in lifespan modulation. Importantly, control experiments confirmed that baicalein neither inhibited *E. coli* OP50 growth nor altered *C. elegans* feeding behavior, ruling out dietary restriction, a known longevity mechanism in *C. elegans*, as the cause of lifespan extension. Thus, the lifespan effects of baicalein are independent of food intake changes or microbial interactions.

Previous studies have identified the key transcription factor DAF‐16, which acts as a “capacitor” to stabilize the transcriptome during aging and regulates stress and longevity genes [32, 33]. This finding aligns with our observations, including baicalein‐induced enhanced DAF‐16 nuclear GFP fluorescence, coordinated upregulation of *sod‐3*, potential modulation of a broader network of stress‐related genes, and final enhancement of both lifespan and healthspan. *sod‐3* expression is typically low in early adulthood, increases under oxidative stress, and declines with age. It correlates with reduced stress resistance and oxidative damage accumulation [34]. A related study [35] reported an age‐dependent decrease in *sod‐3* expression starting from adult Day 2. This aligns with our finding that *sod‐3* levels peaked on Day 2 and declined thereafter during early adulthood.

While baicalein administration did not significantly alter the lifespan of *skn‐1* mutants in our study, the absence of SKN‐1 nuclear translocation and downstream gene activation (*gcs‐1* and *gst‐4*) provides insufficient evidence for SKN‐1 activation. Thus, we cannot conclusively establish SKN‐1 pathway activation as a complete mechanism underlying baicalein‐induced longevity. Furthermore, the pronounced lifespan alterations in *pmk‐1(km25)* mutants suggest potential dysfunction of PMK‐1, the upstream regulator of SKN‐1, which cast additional doubt on SKN‐1 activation. This contrasts with a recent study from Havermann et al. [18], which posits a role for *skn‐1* in baicalein‐induced longevity [18]. While their work employed *skn‐1* RNAi in *C. elegans* to associate baicalein with enhanced stress resistance and longevity, where incomplete *skn‐1* silencing may compromise the validity of these conclusions. Their follow‐up study [19] utilized SKN‐1::GFP nuclear translocation as a mechanistic indicator but omitted lifespan validation for the proposed longevity effects. The SKN‐1 pathway in C. elegans, analogous to the human Nrf2 system, is crucial for longevity and oxidative stress responses [36]. Guerrero‐Rubio et al. [17] proposed that baicalein extends lifespan via the SKN‐1/Nrf2 pathway but did not account for potential dietary restriction confounders due to *E. coli* inhibition. Consequently, further comprehensive experimental validations are warranted to clarify the role of SKN‐1 in baicalein‐induced longevity.

In the future, transcriptomic studies in baicalein‐treated *C. elegans* could identify novel IIS effectors or alternative longevity pathways, including mitochondrial unfolded protein response [37] or lipid metabolic regulation [38]. Single‐cell approaches may further pinpoint the targeted cell types and spatiotemporal dynamics of baicalein, which can offer higher‐resolution insights into its antiaging mechanisms. For example, enhanced DAF‐16 nuclear translocation in intestinal cells versus hypodermis would suggest tissue‐specific enhancement of metabolic stress resistance. Conversely, neuronal *daf‐2* downregulation without corresponding DAF‐16 activation might implicate non‐cell‐autonomous longevity regulation through inter‐tissue signaling.

Baicalein has shown promise as an antiaging agent in recent in vitro and in vivo studies [18, 19, 39]. Mohanty and Suchiang [39] identified the capacity of baicalein to inhibit the Wnt pathway, which suggests its potential as a therapeutic target in age‐related diseases characterized by aberrant Wnt activation. Furthermore, studies by Havermann et al. [18, 19] linked SKN‐1 activation to increased lifespan. However, translating these findings into human applications remains challenging and necessitates comprehensive clinical studies. Additionally, there is limited information on the long‐term use and toxicity of baicalein. Currently, baicalein is primarily consumed as a supplement, and commercially available supplements are promoted for their anti‐inflammatory, antioxidant, and putative antiaging properties. A study by Dong et al. [40] found that baicalein supplements are safe and well tolerated within the recommended dosage range among healthy Chinese volunteers. And no significant or severe adverse effects were observed. Each baicalein tablet used in this study contained 500 mg of the active compound, with an effective dosage of 100 mg per tablet. Following oral administration of baicalein in varying doses (200–800 mg in 200 mg increments), the compound was rapidly absorbed, with mean peak plasma concentrations of 280.44, 628.80, 845.20, and 489.55 ng/mL, respectively. The time to reach maximum concentration was approximately 3 h for each dose, followed by a gradual decline.

## 4 Conclusion

Taken together, our study provides a comprehensive and methodologically rigorous analysis of the effect of baicalein on *C. elegans* longevity, healthspan, and signaling. Through a wide array of experimental approaches, including lifespan, mobility, lipofuscin accumulation, oxidative stress resistance assays, and RNA and protein expression analyses, we have demonstrated the potential of baicalein as an antiaging compound. The applied methodologies, which included Kaplan‐Meier survival analysis, live imaging, video recording, fluorescence microscopy, and real‐time PCR, alongside advanced genetic and biochemical techniques, underscore the utility of *C. elegans* as a model organism for aging studies. The insights gained from our results may pave the way for further exploration into the molecular mechanisms underlying aging and the development of strategies to extend lifespan and enhance healthspan.

## 5 Materials and Methods

### 5.1 *C. elegans* Maintenance, Synchronization, and Drug Administration

*C. elegans* strains including wild‐type N2, CF1038, CB1370, GR1309, EU1, KU25, VC199, DA1116, TJ356, CF1553, and LD1 were obtained from the *Caenorhabditis* Genetics Center (CGC) (University of Minnesota, Minneapolis, USA). Strain information is summarized in Table S3. *C. elegans* was maintained on NGM plates containing live *E. coli* OP50, which were treated with 100 µM baicalein, a concentration selected based on prior studies [18, 19]. Briefly, baicalein (Sigma–Aldrich, Steinheim, Germany) was dissolved in DMSO (AppliChem, Darmstadt, Germany) to generate a 100 mM stock solution, which was diluted in live OP50 liquid culture to the working concentration. While control groups received equivalent 0.1% DMSO. Age‐synchronized nematodes were generated by transferring gravid adults onto seeded NGM plates for a 2‐h egg‐laying period. Following adult removal, eggs were maintained for hatching under standard conditions prior to experimental use in subsequent assays.

### 5.2 Determination of the Baicalein Inhibitory Effect on *E. coli* OP50 Growth

The *E. coli* OP50 growth exposed to 100 µM baicalein was assessed at 30‐min intervals using a photometer, following a described method [8]. Briefly, 15 mL of OP50 liquid culture (OD_595_ = 0.1) containing either 100 µM baicalein or equivalent 0.1% DMSO were prepared. Both samples were then shaken at 220 rpm at 37°C, with OD_595_ measurements recorded at 30‐min intervals over 8 h. To further assess bacterial growth, both groups were incubated at 37°C, with OD_595_ measurements recorded at the 24th and 48th hours. Each of the assays had three independent biological repeats.

### 5.3 Lifespan Assay

Lifespan assays followed established protocols [41]. Synchronized nematodes developed from egg to late L4 stage (Day 0) within approximately 40 h. Nematodes unresponsive to tactile stimuli were scored as dead and removed. Missing individuals or internal hatching were censored, with all abnormalities documented. Experiments terminated upon full mortality. The assays for every strain/group had three independent biological repeats.

### 5.4 Bacteria Avoidance Assay

Bacterial avoidance assays used 6 cm NGM plates bisected into halves: one with OP50 + 0.1% DMSO (control) and the other with OP50 + 100 µM baicalein (Figure 2C) [8]. Fifty nematodes were centrally placed, with distribution across halves recorded at 3‐ and 6‐h intervals to assess avoidance behavior. Each of the assays had four independent biological repeats.

### 5.5 Assays of Body Bending and Pharyngeal Pumping

Body bending assays followed established protocols [8, 42]. Age‐synchronized wild‐type N2 nematodes were assessed on Days 6/9/12, *n* = 20 (20°C, NGM plates with 0.1% DMSO or 100 µM baicalein). Nematodes were transferred to unseeded plates for unrestricted movement. After 1–2 min acclimation, body bends were counted for 1 min under a Nomarski microscopy.

Pharyngeal pumping assays followed protocols [41, 43]. N2 nematodes were treated as above. On Days 9/12, 20 acclimated nematodes were observed under a Nomarski microscopy to record terminal bulb contractions. The pumps were counted at a slower playback of the recorded video.

### 5.6 Levels of Lipofuscin and Intracellular ROS

Lipofuscin assays followed established protocols [8]. Age‐synchronized N2 nematodes were maintained as above. On Day 12, 20 nematodes per group were washed with M9 buffer three times, mounted on 2% agar pads, and immobilized with 10 mM sodium azide (Merck, Darmstadt, Germany). Gut‐focused images were captured via fluorescence microscopy with excitation wavelengths of 340–390 nm and emission wavelengths producing blue light between 430 and 490 nm under standardized 1000 ms exposure [44]. Lipofuscin levels (fluorescence intensity) were quantified using ImageJ (version 1.53t).

Superoxide levels were assessed via DHE staining, metabolizing into 2‐EOH by superoxide [45]. On Day 10, N2 nematodes (*n* = 20) were dark‐incubated with 5 µM DHE (1 h, 20°C), washed, and immobilized on agar pads with sodium azide. The ROS fluorescence, excited around 518 nm and emitting red fluorescence at 605 nm, was imaged at 1000 ms exposure using a fluorescence microscope. Fluorescence intensity, reflecting superoxide levels, was quantified with ImageJ.

### 5.7 Total RNA Isolation and Real‐Time PCR

Total RNA was extracted from 200 age‐synchronized N2 nematodes per group (Days 1–3) using TRIzol (Ambion by Life Technologies, Carlsbad, USA) and the RNeasy Mini Kit (QIAGEN, Hilden, Germany) following manufacturer protocols. RNA (1 µg) was reverse‐transcribed with the High‐Capacity RNA‐to‐cDNA Kit (Thermo Fisher Scientific Baltics UAB, Vilnius, Lithuania). Real‐time PCR was performed on a StepOne System (Applied Biosystems, California, USA) using PowerUp SYBR Green Master Mix (Thermo Fisher Scientific Baltics UAB) and primers sourced from Eurofins Genomics, Ebersberg, Germany as shown in Table 1.

**TABLE 1:** Real‐time PCR primers.

Gene	Annotation	Primer pair
*act‐1*	Actin‐related gene (Housekeeping gene)	FWD: CCAGGAATTGCTGATCGTATGCAGAA REV: TGGAGAGGGAAGCGAGGATAG
*sod‐3*	Superoxide dismutase	FWD: CACACTCTCCCAGATCTCCC REV: AATTTCAGCGCTGGTTGGAG
*mtl‐1*	Metallothionein	FWD: GGAGGCCAGTGAGAAAAAATG REV: GCTTCTGCTCTGCACAATGAC
*dod‐3*	Downstream of DAF‐16 (regulated by DAF‐16)	FWD: GGAGTCCTGCTCTCAGATGAA REV: ACATGAACACCGGCTCATTC
*gst‐4*	Glutathione S‐transferase	FWD: CCCATTTTACAAGTCGATGG REV: CTTCCTCTGCAGTTTTTCCA
*ctl‐1*	Catalase	FWD: TCGTTCATGCCAAGGGAGC REV: GATCCCGATTCTCCAGCGAC
*ctl‐2*	Catalase	FWD: GAAGGTGTTGGATACCGGGG REV: GGATGAGTGCCTTGACACGA
*gcs‐1*	Gamma‐glutamylcysteine synthetase	FWD: AATCGATTCCTTTGGAGACC REV: ATGTTTGCCTCGACAATGTT

Primer sequences were chosen according to existing publications [8, 28, 47].

### 5.8 Quantitation of SOD‐3 Expression

SOD‐3::GFP assays followed established protocols [8]. Age‐synchronized CF1553 nematodes were treated as above. On Day 2 (adult stage) or Day 4 posttreatment, 20 nematodes per group were washed, immobilized with 5 mM levamisole (Sigma–Aldrich, St. Louis, USA) on 2% agar pads, and imaged via fluorescence microscopy the excitation at 488 nm, emission between 500 and 530 nm (3000 ms exposure). Fluorescence intensity was quantified using ImageJ.

### 5.9 Intracellular Localization of DAF‐16 and SKN‐1

SKN‐1 and DAF‐16 nuclear localization was analyzed using established methods [46]. The DAF‐16::GFP reporter strain TJ356 was treated with 100 µM baicalein to assess nuclear translocation [33]. Age‐synchronized nematodes underwent standard treatment. And on Day 2 of adulthood, 20 nematodes were washed with M9 buffer, anesthetized with 5 mM levamisole hydrochloride, and imaged on 2% agar pads. Fluorescence distribution was examined with 3000 ms exposure, focusing on nuclear (vs. cytoplasmic) GFP signals. Similarly, SKN‐1 localization was evaluated using LD1 strain expressing SKN‐1::GFP under identical conditions and imaged daily during early adulthood. Both experiments employed blinded analysis using a nuclear/cytoplasmic (n/c) GFP intensity ratio: cytoplasmic (<1.2), weakly nuclear (1.2–2.0), and strongly nuclear (>2.0) [33].

### 5.10 Statistical Analysis

Data are presented as mean ± standard deviation (SD). Group comparisons used Student's *t*‐test, while DAF‐16::GFP localization effects of 100 µM baicalein were analyzed by Fisher's exact or chi‐square tests. *C. elegans* lifespan was assessed via log‐rank (Mantel–Cox) test using Prism 10.1.2. Significance (*p < 0.05, **p < 0.01) was determined at *p *< 0.05.

## Author Contributions

Chen Zhao: Concept and design, acquisition of data, analysis and interpretation of data, analysis and interpretation of data, and interpretation of data. Chen Zhao and Daniel Schrapel: Development of methodology. Michael Schäfer: Technical support. All authors have read and approved the final manuscript.

## Funding Information

The authors have nothing to report.

## Ethics Statement

The authors have nothing to report.

## Conflicts of Interest

All authors declared no conflicts of interest.

## Supporting information




**Supporting Table 1**: Outcomes of three independent lifespan assays on wild‐type N2 nematode strain and statistical analysis.
**Supporting Table 2**: Individual results of lifespan assays for each of three experiments.
**Supporting Table 3**: Information on *C. elegans* wild‐type N2 and mutant strains.
**Supporting Figure 1**: Lacking evidence for baicalein‐induced SKN‐1 nuclear translocation (A) A *skn‐1::gfp* reporter construct was present in the age‐synchronized hatched larvae (L1 stage) of the *C. elegans* LD1 strain, which were cultivated on NGM agar plates either with 100 µM baicalein (BC) or 0.1% DMSO as a control (CO). On adult Days 1, 2, and 3, 20 nematodes per condition were captured by a microscope with excitation near 488 nm and emission at 500‐530 nm and typical photos for each condition of each day are displayed. (B) Total fluorescence of each condition was measured and data are mean ± SD. (*p* > 0.05).
**Supporting Figure 2**: Survival curves of all the lifespan experiments in supplementary table S1.
**Supporting Figure 3**: Survival curves of all the lifespan experiments in supplementary table S2.
**Supporting Figure 4**: Dissociation curves of eight genes including gcs‐1, dod‐3, ctl‐1, ctl‐2, gst‐4, sod‐3, act‐1 and mtl‐1 at three time points (adult Day 1, adult Day 2 and adult Day 3) of 200 worms treated with baicalein with three trials.
**Supporting Figure 5**: Validation of the primers' effectiveness (sod‐3).

## Data Availability

All data that support the findings of this study are available in the manuscript and its supplement or are available from the corresponding author upon request.
