# Supplementary material for: Baicalein Enhances Longevity and Healthspan of C. elegans Through the Insulin/IGF‐1 Signaling Pathway
Source: MedComm (2020). 2025 Dec 17;6(12):e70543. doi: 10.1002/mco2.70543 (PMC12710425; doi:10.1002/mco2.70543)
Supplement: Supplementary file 1 — Supporting Table 1: Outcomes of three independent lifespan assays on wild‐type N2 nematode strain and statistical analysis. Supporting Table 2: Individual results of lifespan assays for each of three experiments. Supporting Table 3: Information on C. elegans wild‐type N2 and mutant strains. Supporting Figure 1: Lacking evidence for baicalein‐induced SKN‐1 nuclear translocation (A) A skn‐1::gfp reporter construct was present in the age‐synchronized hatched larvae (L1 stage) of the C. elegans LD1 strain, which were cultivated on NGM agar plates either with 100 µM baicalein (BC) or 0.1% DMSO as a control (CO). On adult Days 1, 2, and 3, 20 nematodes per condition were captured by a microscope with excitation near 488 nm and emission at 500‐530 nm and typical photos for each condition of each day are displayed. (B) Total fluorescence of each condition was measured and data are mean ± SD. (p > 0.05). Supporting Figure 2: Survival curves of all the lifespan experiments in supplementary table S1. Supporting Figure 3: Survival curves of all the lifespan experiments in supplementary table S2. Supporting Figure 4: Dissociation curves of eight genes including gcs‐1, dod‐3, ctl‐1, ctl‐2, gst‐4, sod‐3, act‐1 and mtl‐1 at three time points (adult Day 1, adult Day 2 and adult Day 3) of 200 worms treated with baicalein with three trials. Supporting Figure 5: Validation of the primers' effectiveness (sod‐3). [file MCO2-6-e70543-s001.docx]

**Supplementary materials**

**Baicalein enhances longevity and healthspan of**

***C. elegans* through the insulin/IGF-1 signaling pathway**

**Chen Zhao^1,2,*^, Daniel Schrapel^2^, Michael Schaefer^2^**

^1^ Department of Pediatrics, Union Hospital, Tongji Medical College, Huazhong University of Science and Technology, Wuhan, China

^2^ Molecular OncoSurgery, Section Surgical Research, Department of General, Visceral & Transplant Surgery, University of Heidelberg, Heidelberg, Germany

**^*^Corresponding author**

[zhaochen2015@hust.edu.cn](mailto:zhaochen2015@hust.edu.cn)

**Supplementary table S1. Outcomes of three independent lifespan assays on wild-type N2 nematode strain and statistical analysis**

| **Strain** | **Exp.**  **No.** | **Treatment** | **Nematode**  **No.** | **Death**  **events** | **Censored**  **subjects** | **Median survival**  **(days)** | **Life extension ratio (95% CI)** | ***p*-value**  **vs. control** |
| --- | --- | --- | --- | --- | --- | --- | --- | --- |
| N2 | 1 | CO | 123 | 77 | 46 | 20 | 115%  (84% ~ 158%) | 0.039 |
|  |  | BC | 120 | 76 | 44 | 23 |  |  |
|  | 2 | CO | 120 | 95 | 25 | 20 | 105%  (78% ~ 142%) | 0.034 |
|  |  | BC | 120 | 77 | 43 | 21 |  |  |
|  | 3 | CO | 120 | 86 | 34 | 20 | 110%  (81% ~ 150%) | 0.002 |
|  |  | BC | 120 | 75 | 45 | 22 |  |  |

CO: control, BC: baicalein. Summary of the details of death events, censored subjects, median survival and comparisons for lifespan assays of the wild-type N2 nematode strain. The log-rank (Mantel-Cox) test was used to calculate the median survival. *p*-values were calculated for the control and treatment groups in every trial. The difference in longevity between the two groups was statistically significant (*p* < 0.05).

**Supplementary table S2. Individual results of lifespan assays for each of three experiments**

| **Strain/**  **mutated gene** | **Temp.** | **Exp.**  **No.** | **Trea-**  **tment** | **Nematode**  **No.** | **Death**  **events** | **Censored**  **subjects** | **Median survival**  **(days)** | **Life extension ratio**  **(95% CI)** | ***p*-value**  **vs.**  **control** |
| --- | --- | --- | --- | --- | --- | --- | --- | --- | --- |
| CF1038  *daf-16(mu86) I* | 20℃ | 1 | CO | 250 | 181 | 69 | 16 | 100%  (81% ~ 123%) | 0.715 |
|  |  |  | BC | 250 | 181 | 69 | 16 |  |  |
|  |  | 2 | CO | 188 | 150 | 38 | 12 | 100%  (81% ~ 124%) | 0.368 |
|  |  |  | BC | 208 | 179 | 29 | 12 |  |  |
|  |  | 3 | CO | 100 | 55 | 45 | 19 | 89%  (62% ~ 129%) | 0.081 |
|  |  |  | BC | 100 | 59 | 41 | 17 |  |  |
| CB1370  *daf-2(e1370) III* | 15℃ | 1 | CO | 207 | 137 | 70 | 44 | 95%  (76% ~ 120%) | 0.882 |
|  |  |  | BC | 224 | 154 | 70 | 42 |  |  |
|  |  | 2 | CO | 110 | 79 | 31 | 45 | 93%  (69% ~ 127%) | 0.670 |
|  |  |  | BC | 113 | 87 | 26 | 42 |  |  |
|  |  | 3 | CO | 110 | 83 | 27 | 45 | 91%  (66% ~ 125%) | 0.411 |
|  |  |  | BC | 110 | 72 | 38 | 41 |  |  |
| GR1309  *daf-16(mgDf47) I; daf-2(e1370) III* | 20℃ | 1 | CO | 245 | 180 | 65 | 16 | 94%  (77% ~ 115%) | 0.627 |
|  |  |  | BC | 249 | 193 | 56 | 15 |  |  |
|  |  | 2 | CO | 100 | 59 | 41 | 14 | 93%  (65% ~ 132%) | 0.598 |
|  |  |  | BC | 100 | 67 | 33 | 13 |  |  |
|  |  | 3 | CO | 80 | 57 | 23 | 15 | 107%  (74% ~ 153%) | 0.520 |
|  |  |  | BC | 80 | 60 | 20 | 16 |  |  |
| EU1  *skn-1(zu67) IV/nT1 [unc-?(n754) let-?] (IV;V)* | 20℃ | 1 | CO | 150 | 91 | 59 | 20 | 95%  (70% ~ 128%) | 0.309 |
|  |  |  | BC | 150 | 80 | 70 | 19 |  |  |
|  |  | 2 | CO | 160 | 144 | 16 | 13 | 100%  (80% ~ 125%) | 0.084 |
|  |  |  | BC | 171 | 157 | 14 | 13 |  |  |
|  |  | 3 | CO | 80 | 49 | 31 | 14 | 100%  (67% ~ 149%) | 0.960 |
|  |  |  | BC | 85 | 47 | 38 | 14 |  |  |
| KU25  *pmk-1(km25) IV* | 20℃ | 1 | CO | 150 | 94 | 56 | 15 | 113%  (84% ~ 152%) | 0.007 |
|  |  |  | BC | 150 | 83 | 67 | 17 |  |  |
|  |  | 2 | CO | 100 | 65 | 35 | 14 | 114%  (81% ~ 161%) | 0.019 |
|  |  |  | BC | 100 | 65 | 35 | 16 |  |  |
|  |  | 3 | CO | 148 | 76 | 72 | 16 | 106%  (77% ~ 146%) | 0.030 |
|  |  |  | BC | 148 | 74 | 74 | 17 |  |  |
| VC199  *sir-2.1(ok434) IV* | 20℃ | 1 | CO | 174 | 129 | 45 | 16 | 113%  (88% ~ 143%) | < 0.0001 |
|  |  |  | BC | 179 | 133 | 46 | 18 |  |  |
|  |  | 2 | CO | 120 | 91 | 29 | 20 | 110%  (83% ~ 147%) | 0.0001 |
|  |  |  | BC | 125 | 96 | 29 | 22 |  |  |
|  |  | 3 | CO | 110 | 80 | 30 | 19 | 105%  (77% ~ 143%) | 0.025 |
|  |  |  | BC | 110 | 82 | 28 | 20 |  |  |
| DA1116  *eat-2(ad1116) II* | 20℃ | 1 | CO | 110 | 90 | 20 | 25 | 112%  (83% ~ 151%) | 0.015 |
|  |  |  | BC | 110 | 85 | 25 | 28 |  |  |
|  |  | 2 | CO | 160 | 101 | 59 | 19 | 121%  (91% ~ 160%) | 0.0004 |
|  |  |  | BC | 160 | 94 | 66 | 23 |  |  |
|  |  | 3 | CO | 119 | 69 | 50 | 20 | 120%  (88% ~ 165%) | 0.005 |
|  |  |  | BC | 127 | 88 | 39 | 24 |  |  |

CO: control, BC: baicalein. Summary of the details of deaths events, censored subjects, median survival and comparisons for lifespan assays of *C. elegans* mutant strains. Using the log-rank (Mantel-Cox) test, the median survival was calculated. *p*-values were calculated for each trial that included treatment and control groups. For the populations in the control and baicalein groups, a significant difference in the lifespan assay is shown by *p* < 0.05, and vice versa.

**Supplementary table S3. Information on *C. elegans* wild-type N2 and mutant strains**

| **Strain name**  **WormBase ID**  **Genotype** | **Description** | **References** |
| --- | --- | --- |
| N2  00000001  Wild-type | Its function as a key biological model in genetic and developmental biology was established when Sydney Brenner isolated it in the 1960s. Its life cycle lasts around three days at 20°C, and its average brood size is 330 eggs. It exhibits the typical activities of *C. elegans*, including as eating, thrashing in liquid, and sinusoidal crawling on solid surfaces. | ^1,2^ |
| CF1038  00004840  *daf-16(mu86) I* | FOXO4 is the human homologue gene for *daf-16*. created by Cynthia Kenyon using UV mutagenesis combined with TMP. This strain has the allele *daf-16(mu86)*. It has shorter lifespans and dauer formation defects. | ^3,4^ |
| CB1370  00004309  *daf-2(e1370) III* | IGF-1 receptor and insulin receptor are human homologs for *daf-2*. By using EMS mutagenesis, this mutation was created. Reduced aging and increased lifespan are characteristics of this strain. It greatly affects the IIS pathway and is dauer-constitutive, temperature-sensitive, and best maintained at 15°C. | ^5,6^ |
| GR1309  00007897  *daf-16(mgDf47) I; daf-2(e1370) III* | A significant genetic change is present in the double mutants DAF-16 (mgDf47) and DAF-2 (e1370). mgDf47 completely suppresses the dauer-constitutive phenotype of *daf-2* by deleting around 8 kb of the *daf-16* gene, beginning just after exon 4. Shoshanna Gottlieb created this mutation by using γ-irradiation. | ^7,8^ |
| EU1  00007249  *skn-1(zu67) IV/nT1 [unc-?(n754) let-?] (IV;V)* | An array of biological activities, including the formation of endoderm, the endoplasmic reticulum unfolded protein response, and the positive control of macromolecule metabolism, depend on the *skn-1* gene, a homolog of human Nrf2. Bruce Bowerman used EMS mutagenesis to insert mutations into *skn-1*. In addition to producing three different types of offspring, heterozygotes also exhibit an uncoordinated phenotype (UNC), or wild-type (WT), which only produces non-viable eggs, and non-viable eggs directly. UNC worms are chosen carefully in order to maintain the strain. | ^9-11^ |
| KU25  00024040  *pmk-1(km25) IV* | The *pmk-1* gene is a homolog of human MAPK11 and MAPK14 and is a member of the p38 Map kinase family. Through UV/TMP mutagenesis, Kunihiro Matsumoto produced this mutant. It displays oxidative stress and heavy metal sensitivity. | ^12,13^ |
| VC199  00035578  *sir-2.1(ok434) IV* | The *sir-2.1* gene is a homolog of the human sirtuin 1. Although it is a mutant strain, B. J. Allan produced it via UV/TMP mutagenesis, and it exhibits wild-type phenotypes. | ^14^ |
| DA1116  00005548  *eat-2(ad1116) II* | A key component of pharyngeal pumping is the *eat-2* gene of *C. elegans*, which is orthologous to human CHRFAM7A and CHRNA7. With the use of EMS mutagenesis, David Raizen altered this gene. | ^15^ |
| TJ356  00034892  *zIs356 [daf-16p::daf-16a/b::gfp + rol-6(su1006)]* | By using γ-irradiation, the *daf-16* gene was created by the A. Rougvie lab and connected to a *gfp* (green fluorescent protein) reporter gene under the control of the *daf-16* promoter. | ^16^ |
| CF1553  00004861  *muIs84 [(pAD76) sod-3p::gfp + rol-6(su1006)]* | In the head, tail, and vulva regions, the *sod-3* gene exhibits green fluorescence. It is controlled by its own promoter and is connected to a *gfp* reporter. Even when certain animals don't roll at all, the *gfp* expression is still present. | ^17,18^ |
| LD1  00024125  *ldIs7 [skn-1b/c::gfp + rol-6(su1006)]* | The C-terminus of SKN-1 is fused with GFP. Using a UV/TMP mutagen, Jae Hyung An created this. | ^19^ |

**
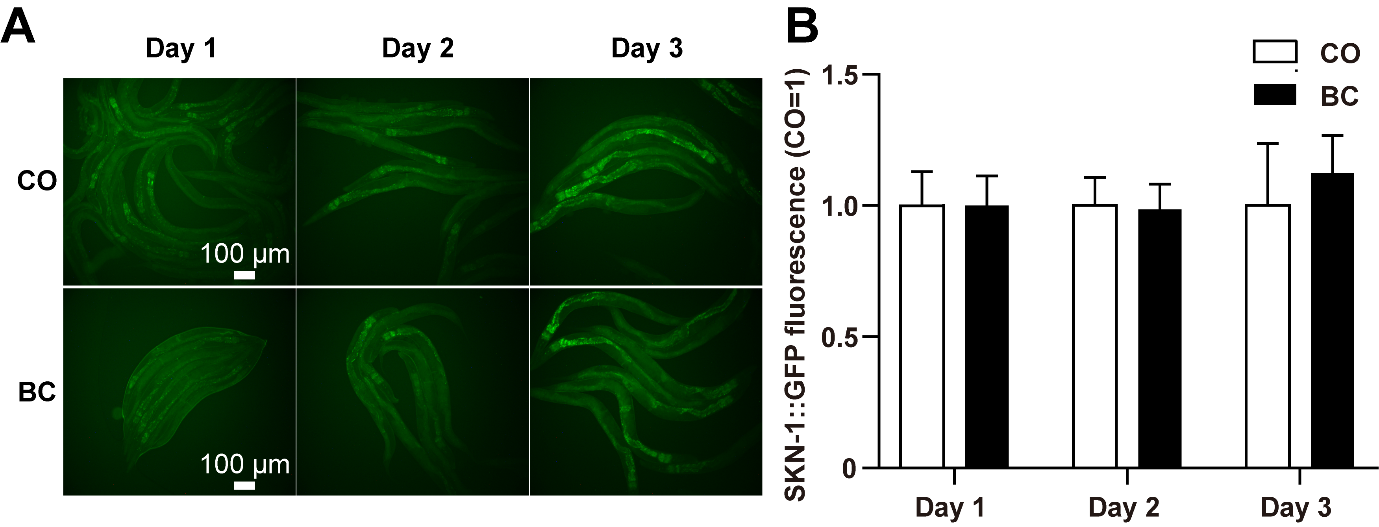
**

**Supplementary figure S1. Lacking evidence for baicalein-induced SKN-1 nuclear translocation** (A) A *skn-1::gfp* reporter construct was present in the age-synchronized hatched larvae (L1 stage) of the *C. elegans* LD1 strain, which were cultivated on NGM agar plates either with 100 µM baicalein (BC) or 0.1% DMSO as a control (CO). On adult days 1, 2, and 3, 20 nematodes per condition were captured by a microscope with excitation near 488 nm and emission at 500-530 nm and typical photos for each condition of each day are displayed. (B) Total fluorescence of each condition was measured and data are mean ± SD. (*p* > 0.05)


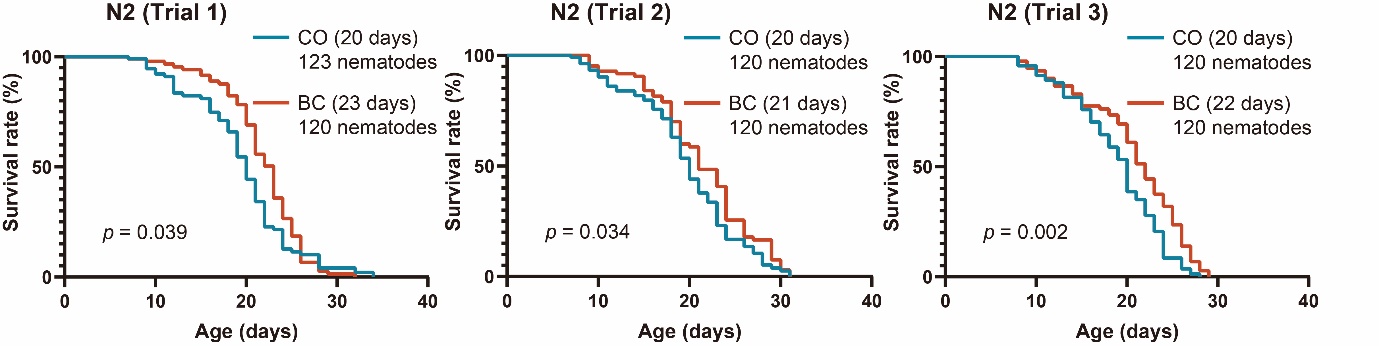


**Supplementary figure S2.** **Survival curves of all the lifespan experiments in supplementary table S1.**


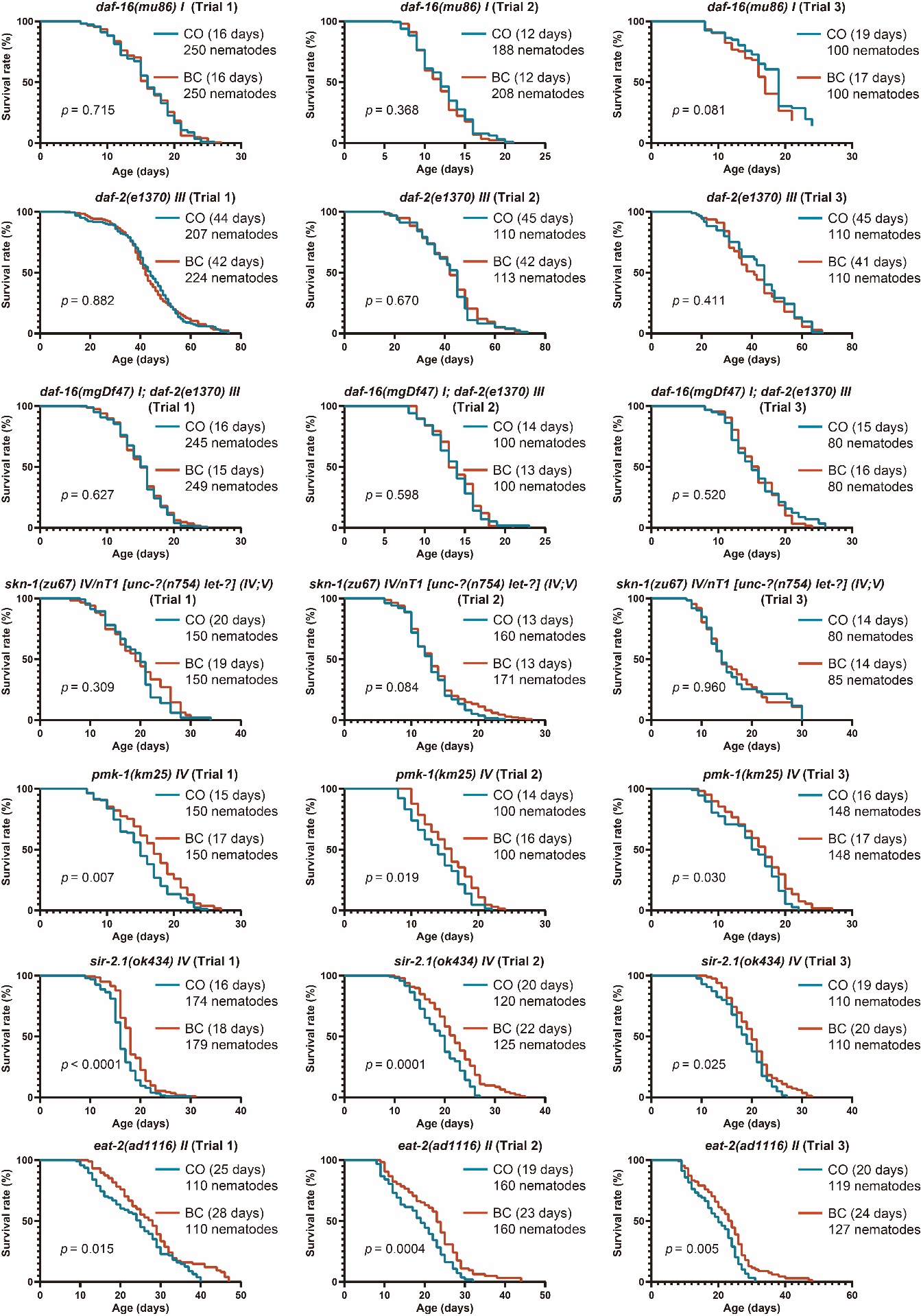


**Supplementary figure S3.** **Survival curves of all the lifespan experiments in supplementary table S2.**


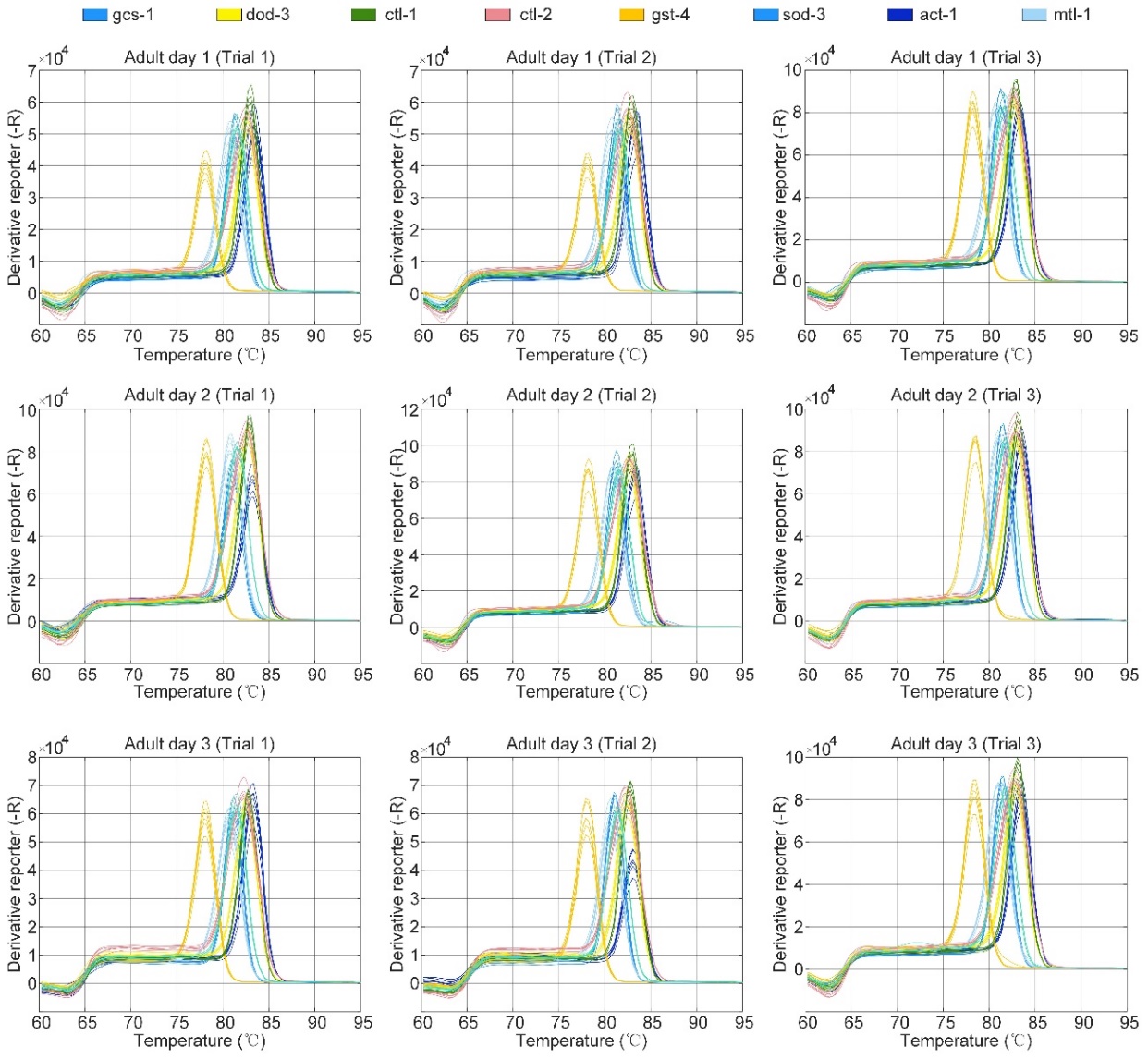


**Supplementary figure S4. Dissociation curves of eight genes including gcs-1, dod-3, ctl-1, ctl-2, gst-4, sod-3, act-1 and mtl-1 at three time points (adult day 1, adult day 2 and adult day 3) of 200 worms treated with baicalein with three trials.**


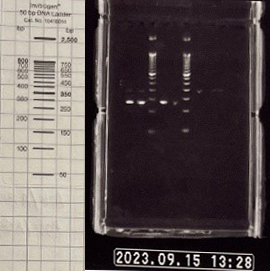


**Supplementary figure S5. Validation of the primers' effectiveness (sod-3).**

**Reference**

1. Nicholas WL. Strain information of N2. <https://cgc.umn.edu/strain/N2>. Published 1951. Accessed February, 2024.

2. Brenner S. The genetics of Caenorhabditis elegans. *Genetics.* 1974;77(1):71-94.

3. Lee SS, Kennedy S, Tolonen AC, Ruvkun G. DAF-16 target genes that control C. elegans life-span and metabolism. *Science (New York, NY).* 2003;300(5619):644-647.

4. Kenyon C, Chang J, Gensch E, Rudner A, Tabtiang R. A C. elegans mutant that lives twice as long as wild type. *Nature.* 1993;366(6454):461-464.

5. Riddle DL, Swanson MM, Albert PS. Interacting genes in nematode dauer larva formation. *Nature.* 1981;290(5808):668-671.

6. Pierce SB, Costa M, Wisotzkey R, et al. Regulation of DAF-2 receptor signaling by human insulin and ins-1, a member of the unusually large and diverse C. elegans insulin gene family. *Genes & development.* 2001;15(6):672-686.

7. Gottlieb S. Strain information of GR1309. <https://cgc.umn.edu/strain/GR1309>. Published 1997. Accessed February, 2024.

8. Lee RY, Hench J, Ruvkun G. Regulation of C. elegans DAF-16 and its human ortholog FKHRL1 by the daf-2 insulin-like signaling pathway. *Current biology : CB.* 2001;11(24):1950-1957.

9. Bowerman B, Eaton BA, Priess JR. skn-1, a maternally expressed gene required to specify the fate of ventral blastomeres in the early C. elegans embryo. *Cell.* 1992;68(6):1061-1075.

10. An JH, Blackwell TK. SKN-1 links C. elegans mesendodermal specification to a conserved oxidative stress response. *Genes & development.* 2003;17(15):1882-1893.

11. Bowerman B. Strain information of EU1. <https://cgc.umn.edu/strain/EU1>. Published 1994. Accessed February, 2024.

12. Berman K, McKay J, Avery L, Cobb MJMcbrc. Isolation and characterization of pmk-(1–3): three p38 homologs in Caenorhabditis elegans. 2001;4(6):337-344.

13. Matsumoto K. Strain information of KU25. <https://cgc.umn.edu/strain/KU25>. Published 2006. Accessed February, 2024.

14. Allan B. Strain information of VC199. <https://cgc.umn.edu/strain/VC199>. Published 2002. Accessed February, 2024.

15. Raizen D. Strain information of DA1116. <https://cgc.umn.edu/strain/DA1116>. Published 1997. Accessed February, 2024.

16. Abbas S, Wink M. Epigallocatechin gallate inhibits beta amyloid oligomerization in Caenorhabditis elegans and affects the daf-2/insulin-like signaling pathway. *Phytomedicine.* 2010;17(11):902-909.

17. Yu X, Su Q, Shen T, Chen Q, Wang Y, Jia W. Antioxidant Peptides from Sepia esculenta Hydrolyzate Attenuate Oxidative Stress and Fat Accumulation in Caenorhabditis elegans. *Mar Drugs.* 2020;18(10).

18. Libina B. Strain information of CF1553. <https://cgc.umn.edu/strain/CF1553>. Published 2004. Accessed February, 2024.

19. An J. Strain information of LD1. <https://cgc.umn.edu/strain/LD1>. Published 2010. Accessed February, 2024.
